# Supplementary material for: Proimmunogenic impact of MEK inhibition synergizes with agonist anti-CD40 immunostimulatory antibodies in tumor therapy
Source: Nat Commun. 2020 May 1;11:2176. doi: 10.1038/s41467-020-15979-2 (PMC7195409; doi:10.1038/s41467-020-15979-2)
Supplement: Supplementary file 1 — Supplementary information [file 41467_2020_15979_MOESM1_ESM.pdf]

Supplementary Information

Baumann et al., 2020

Proimmunogenic impact of MEK inhibition synergizes with agonist anti-CD40 immunostimulatory antibodies in tumor therapy

Supplementary Figure 1

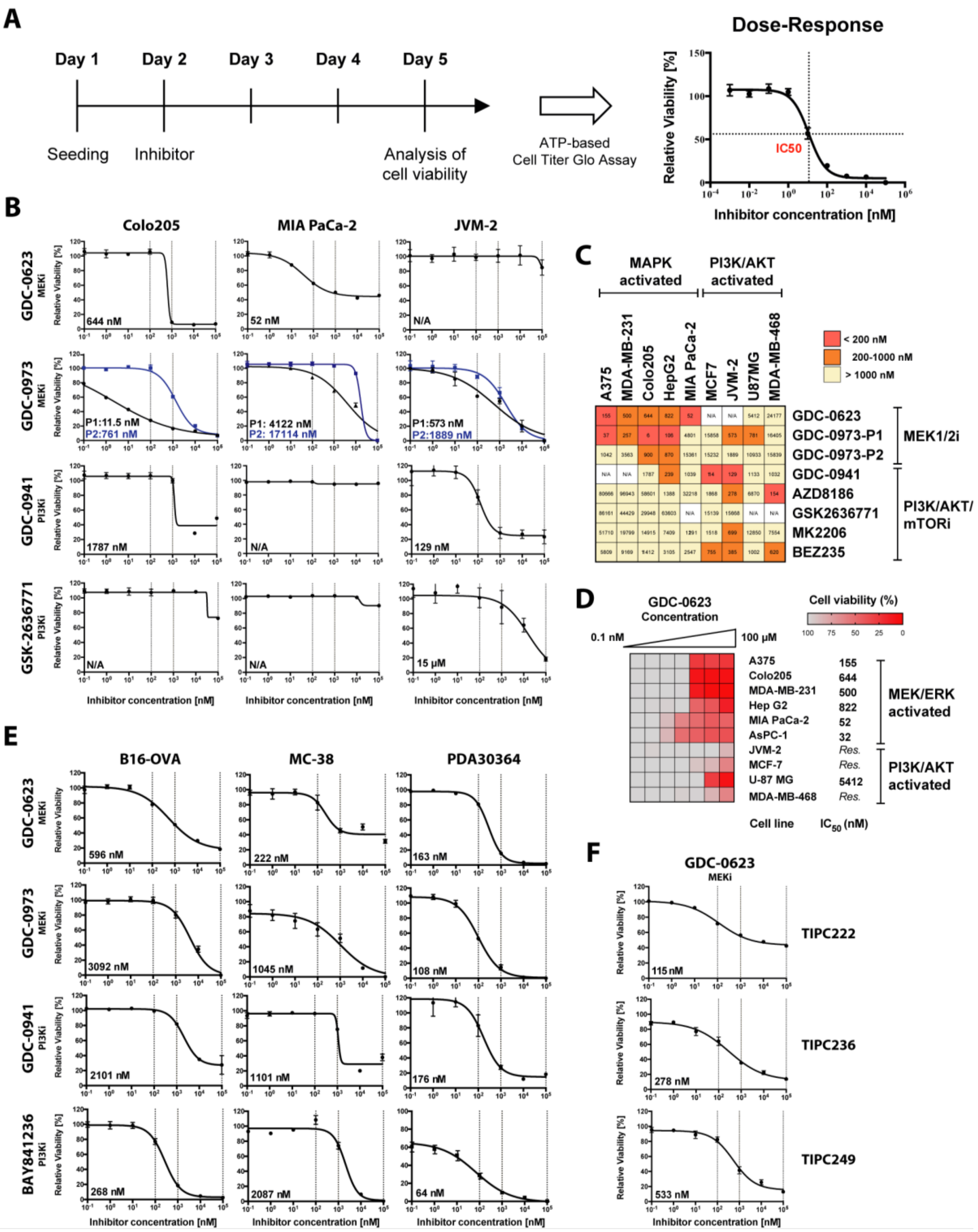

Please see next page for figure legend.

Supplementary Figure 1. In vitro impact of oncology small molecule inhibitors on human reference tumor lines and on T cell proliferation. A Schematic representation of tumor cell viability assay workflow. Relative cell viability was determined by normalization to the medium control. IC50 values were calculated based on a 4-parameter logistic curve fit with a bottom constraint > 0. **B** Representative dose response curves of publicly available human tumor cell lines with activated MAPK or PI3K/AKT signaling treated with MEK and PI3K/AKT/mTOR inhibitors. Mean ± s.e.m, n = 3 (biological replicates from the same experiment). Values depict mean IC50 values of at least two independent experiments. Quality control of BAY compounds on human tumor cell lines was performed by Bayer Pharma AG (Berlin, Germany). **C** Mean IC50 values for various human tumor cell lines treated with indicated small molecule inhibitors. Shading indicates level of inhibitor potency (red: < 200 nM = sensitive; orange: 200-1000 nM = moderate; light yellow: > 1000 nM = not effective). *Res.*: resistant, IC50 > 10 µM; N/A: not available. **D** Dose-responses and mean IC50 values of human tumor cell lines treated with MEK inhibitor GDC-0623. **E** Representative dose response curves of murine tumor cell lines. Mean ± s.e.m, n = 3 (biological replicates from the same experiments). Values depict mean IC50 values of at least two biologically independent experiments. **F** Representative dose response curves of primary PDX PDA cell lines harboring KRAS mutations treated with GDC-0623. Mean ± s.e.m, n = 3. Values depict mean IC50 values of at least two biologically independent experiments.

Supplementary Figure 2

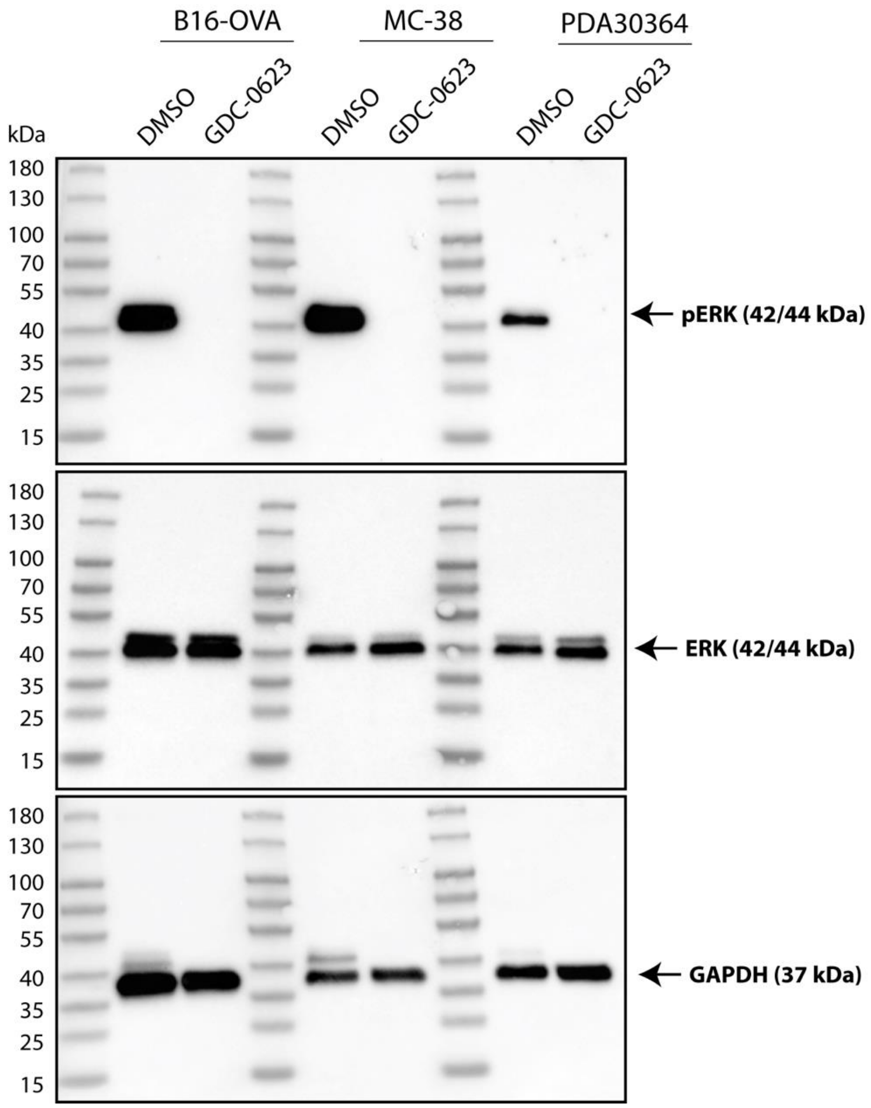

Supplementary Figure 2. Murine tumor cells have high pERK levels. Western Blot analyses of indicated murine tumor cell lines treated with 0.5 µM GDC-0623 or DMSO for 1h.

Supplementary Figure 3

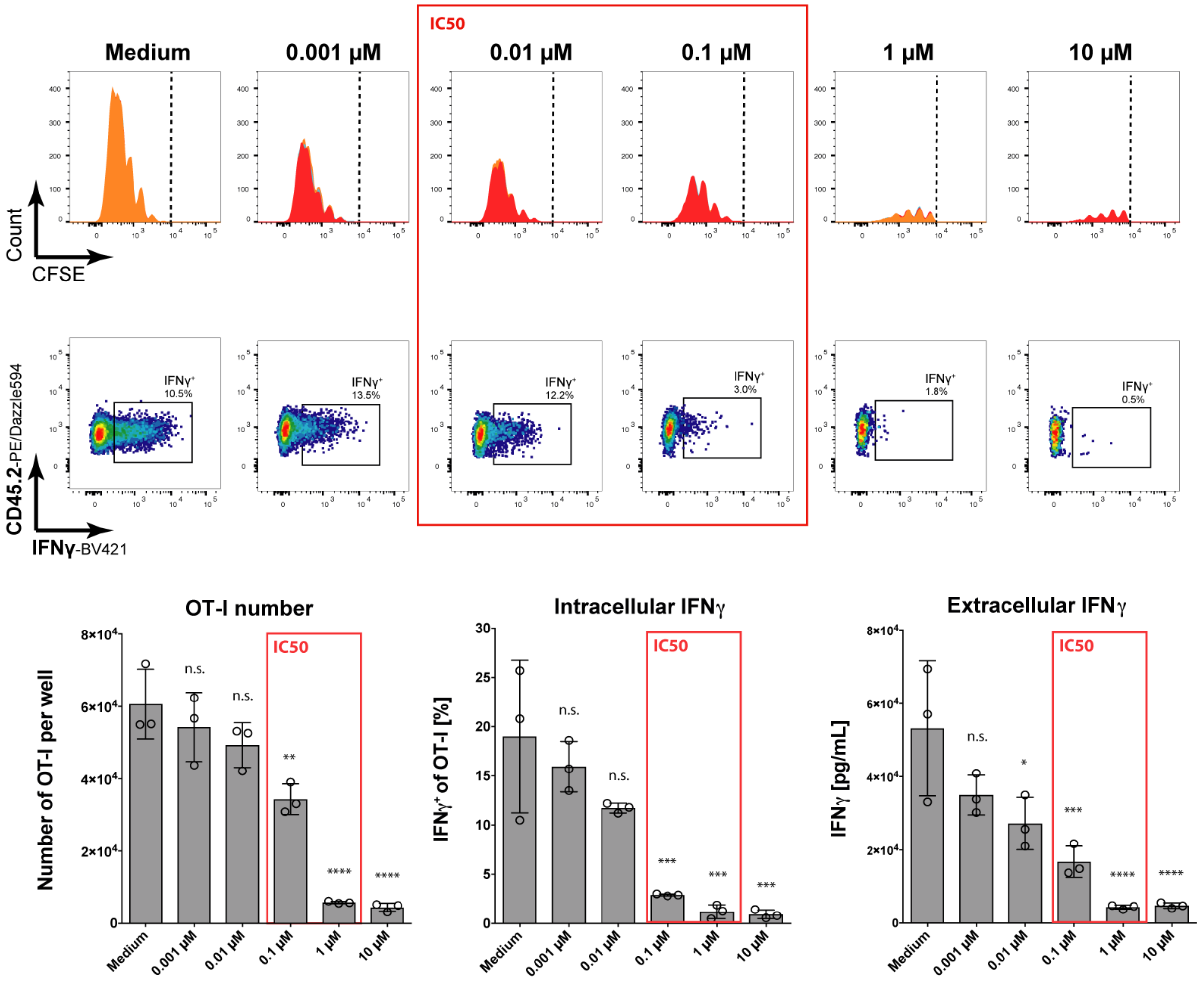

Supplementary Figure 3. MEK inhibitor GDC-0623 suppresses T cell activation and expansion in vitro. Analyses of T cell proliferation and activation in presence of indicated concentration of MEK inhibitor GDC-0623, as determined by CFSE dilution, total T cell numbers, and intracellular flow cytometric-based cytokine staining and supernatant cytokine ELISA. OT-I T cells were co-cultured with MEC.B7.SigOVA antigen presenting cells. Representative of three independent experiments; each experiment comprises three biological replicates, mean ± s.d., n = 3. One-way ANOVA with post-hoc Dunnett's test (treated vs. medium control). P-values OT-I numbers: 0.0012, <0.0001, <0.0001; Intracellular IFNγ: 0.003, <0.0001, <0.0001; Extracellular IFNγ: 0.0842, 0.0009, <0.0001, <0.0001. Significance levels are indicated by asterisks (* $p \leq 0.05$; ** $p \leq 0.01$; *** $p \leq 0.001$; **** $p \leq 0.0001$).

Supplementary Figure 4

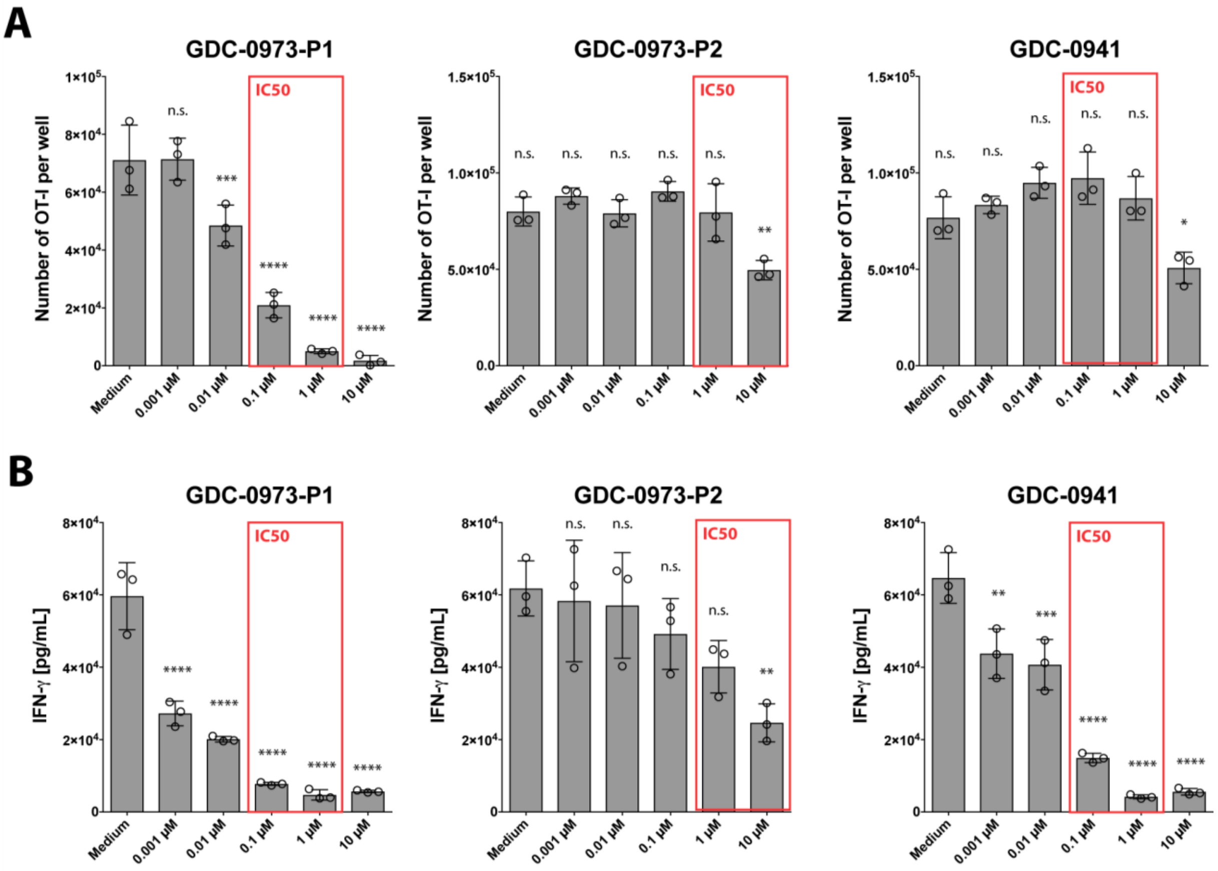

Supplementary Figure 4. MEK and PI3K inhibitors suppress T cells *in vitro*. Analyses of T cell proliferation and activation in presence of indicated concentration of small molecule inhibitors. OT-I T cells were co-cultured with MEC.B7.SigOVA antigen presenting cells. T cell numbers (A) and secreted IFNγ levels (B) were analyzed. Mean ± s.d, n = 3. One-way ANOVA with post-hoc Dunnett's test (treated vs. medium control). P-values OT-I numbers: GDC-0973-P1: <0.0001, <0.0001, <0.0001, 0.0059; GDC-0973-P2: 0.0027; GDC-0941: 0.0277. P-values Extracellular IFNγ: GDC-0973-P1: all <0.0001; GDC-0973-P2: 0.0058; GDC-0941: <0.0001, <0.0001, <0.0001, 0.003, 0.001. Significance levels are indicated by asterisks (* $p \leq 0.05$; ** $p \leq 0.01$; *** $p \leq 0.001$; **** $p \leq 0.0001$).

Supplementary Figure 5

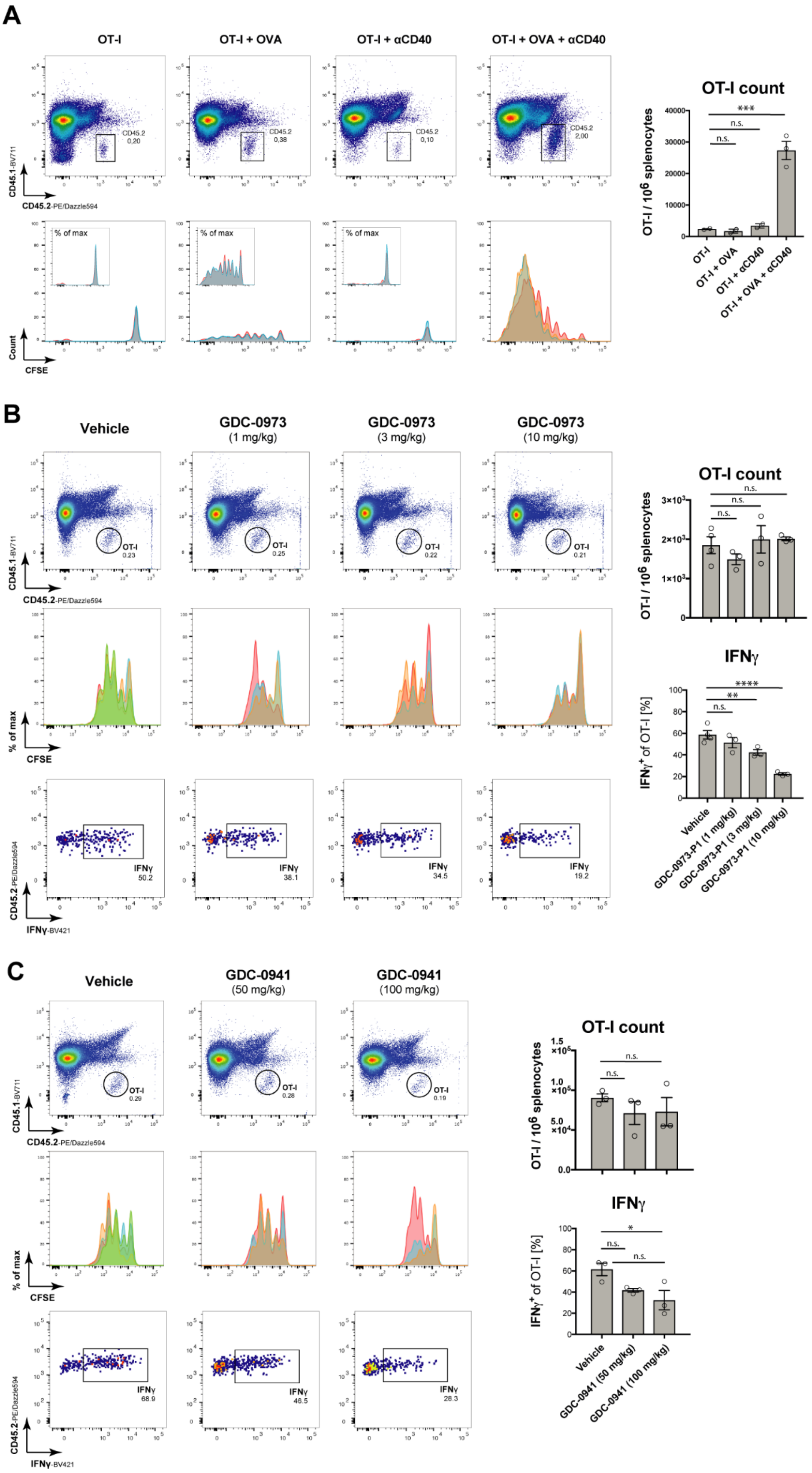

Please see next page for figure legend.

Supplementary Figure 5. In vivo priming of OT-I T cells by whole antigen immunization depends on CD40-mediated costimulation and is only slightly affected by MEK or PI3K inhibition. A *In vivo* OT-I T cell immunization assays. Adoptive transfer of naïve CFSE-labeled OT-I T cells (CD45.2+) via tail vein injection into C57BL/6-Ly5.1 (CD45.1+) mice. After 24 hours, recipients were immunized intravenously with 75 µg OVA protein and 75 µg anti-CD40 mIgG1 Ab or combination. On day five, splenocytes were isolated and T cell proliferation analyzed by flow cytometry. Upper row, dot plots of gated splenic CD45.2+ OT-I T cells from C57BL/6-Ly5.1 (CD45.1+) recipient mice. Lower row, CFSE dilution and re-stimulation with SIINFEKL peptide followed by intracellular IFNγ of gated OT-I T cells; differentially colored histograms represent data from individual mice. Bar charts to the right: quantification of OT-I numbers; n ≥ 3 mice per group. Mean ± s.e.m. P-value OT-I vs. CD40+OVA: 0.0009. The lack of in vivo OT-I T cell expansion after administration of either OVA or anti-CD40 Ab only, as shown in this figure, was verified in multiple experiments and is in line with the published data from Glennie and colleagues, from whom we obtained the anti-CD40 Ab and adapted the assay[1, 2]. **B-C** Host mice were treated daily with the indicated concentrations of MEK inhibitor GDC-0973-P1 or pan-PI3K inhibitor GDC-0941; Bar charts to the right: quantification of OT-I numbers and intracellular IFNγ after ex vivo restimulation; n ≥ 3 mice per group. Mean ± s.e.m. P-value intracellular IFNγ: GDC-0973-P1: 0.0219, 0.0001. GDC-0941: 0.0315. One-way ANOVA with post-hoc Dunnett's test. Significance levels are indicated by asterisks (* p ≤ 0.05; ** p ≤ 0.01; *** p ≤ 0.001; **** p ≤ 0.0001).

Supplementary Figure 6

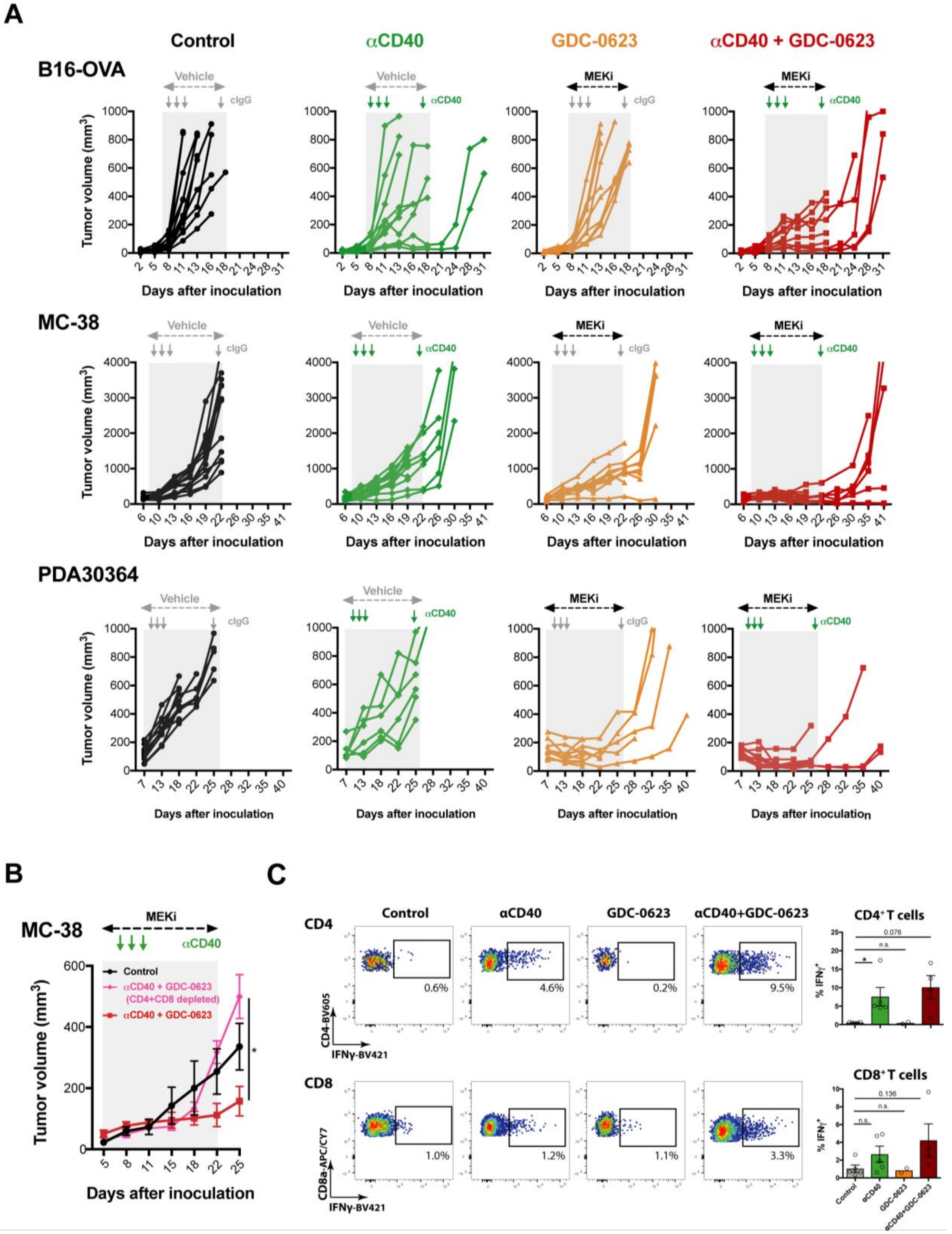

Please see next page for figure legend.

Supplementary Figure 6. Combination of MEK inhibition with agonist anti-CD40 Ab mediates control of established tumors. A Single tumor growth curves of B16-OVA, MC-38, and PDA30364 upon treatment with MEKi/CD40 Ab. Mice were treated daily with 30 mg kg^{-1} GDC-0623 or vehicle solution (MCT) for approximately two weeks (grey rectangle). Anti-CD40 and control Abs, were administered intraperitoneally on treatment days 3, 5, 7 as well as 1 day prior to biomarker analyses (green arrows). Each treatment group consisted of at least 6 animals. **B** T cell depletion in treated MC-38 tumor-bearing animals. Mice were treated daily with 30 mg kg^{-1} GDC-0623 or vehicle solution (MCT) for approximately 10 days (grey rectangle). Anti-CD40 and control Abs were administered intraperitoneally on treatment days 3, 5, 7, 11, 18 and 23 (green arrows). Additional groups received depletory anti-CD4, anti-CD8 or anti-CD4+anti-CD8 Abs twice per week. Each treatment group consisted of at least five mice. Mean ± s.e.m. Two-way ANOVA with post-hoc Tukey's test (day 25). P-value: 0.018. Significance levels are indicated by asterisks (* $p \leq 0.05$). **C** TILs from MC-38 tumors were incubated with Golgi-Plug and stained for intracellular IFNγ. Mean ± s.e.m, n (Control) = 5, n (CD40) = 5, n (GDC-0623) = 2, n (CD40 + GDC-0623) = 4. One-way ANOVA with post-hoc Dunnett's test. P-value CD4: 0.09471. Significance levels are indicated by asterisks (* $p \leq 0.05$; ** $p \leq 0.01$; *** $p \leq 0.001$; **** $p \leq 0.0001$).

Supplementary Figure 7

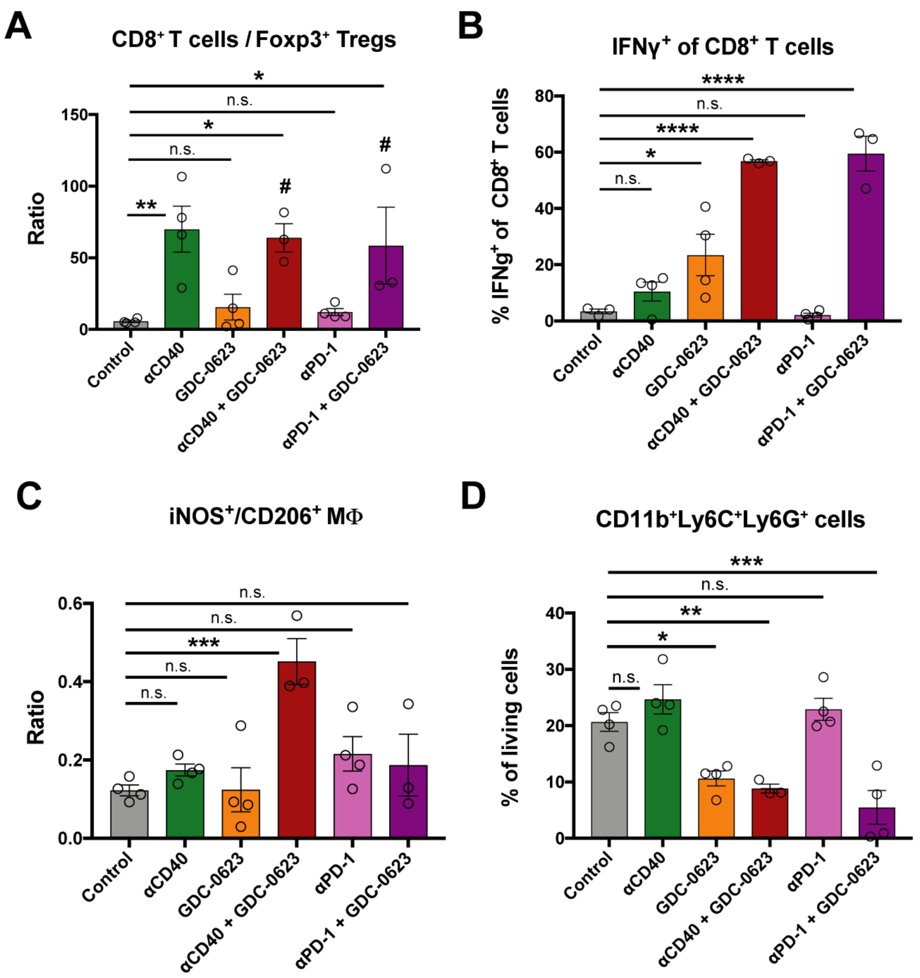

Supplementary Figure 7. Benchmarking of MEKi/anti-CD40 Ab to MEKi monotherapy and MEKi/PD-1 Ab. Mice were treated daily with 30 mg kg^{-1} GDC-0623 or vehicle solution (MCT) for approximately seven weeks. Anti-CD40, anti-PD-1 and control Ab were administered intraperitoneally twice per week. Each treatment group consisted of at least 10 animals. Four mice per group were sacrificed between days 14-16 after treatment start and subjected to biomarker and FACS analyses. **A-D** FACS-based quantification of indicated cell populations in the tumor microenvironment. [#]In both groups one mouse without Tregs. **B** TILs isolated from PDA30364 tumors were restimulated *ex vivo* with PMA/Ionomycin in presence of GolgiPlug and stained for intracellular IFNγ. Mean ± s.e.m. One-way ANOVA with post-hoc Dunnett's test (treatment vs. control; day 22). P-values: (A) 0.0063, 0.0223, 0.0403; (B) 0.02056, 0.0001, 0.0001; (C) 0.0007; (D) 0.0134, 0.0070, 0.0004. Significance levels are indicated by asterisks (* $p \leq 0.05$; ** $p \leq 0.01$; *** $p \leq 0.001$; **** $p \leq 0.0001$).;

Supplementary Figure 8

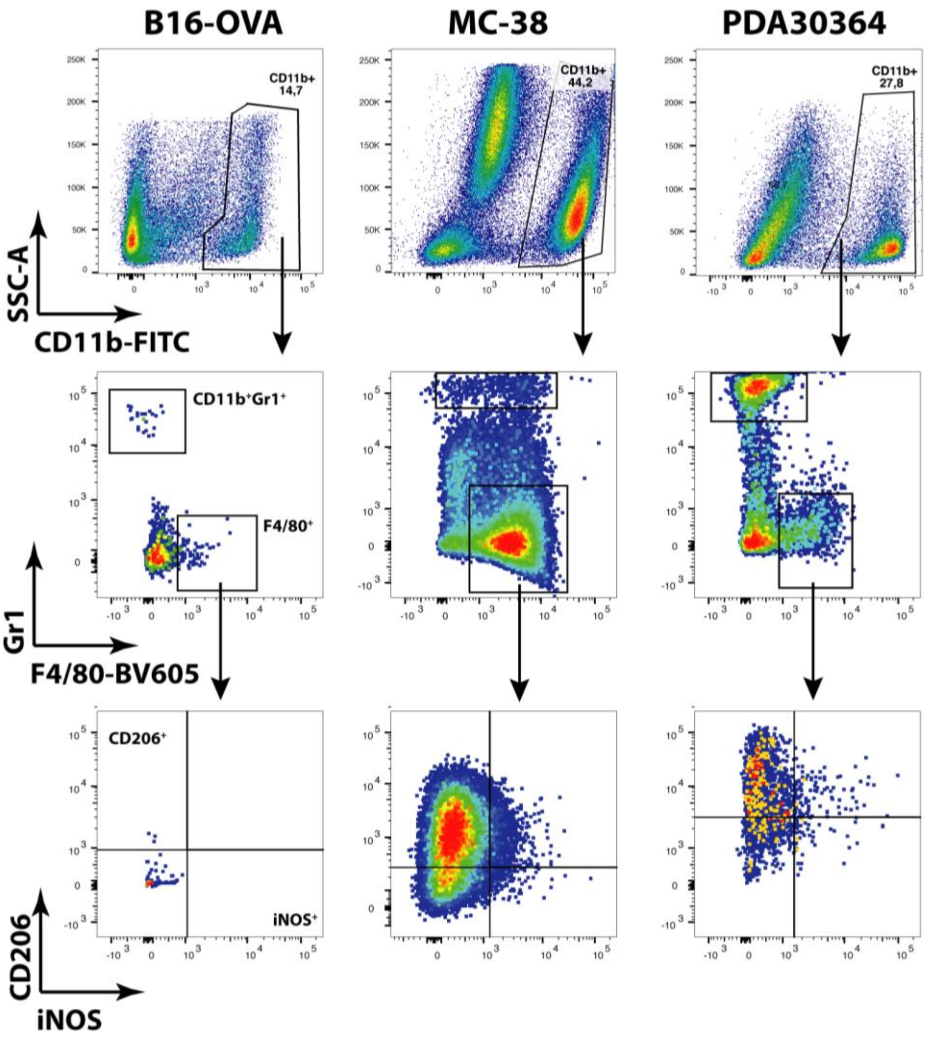

Supplementary Figure 8. Content of myeloid cells in B16-OVA, MC-38 and PDA30364. Flow cytometric analyses of tumor immune cell infiltrate in the indicated models. Representative dot plots of intratumoral myeloid cells. Cells were pre-gated on living cells.

Supplementary Figure 9

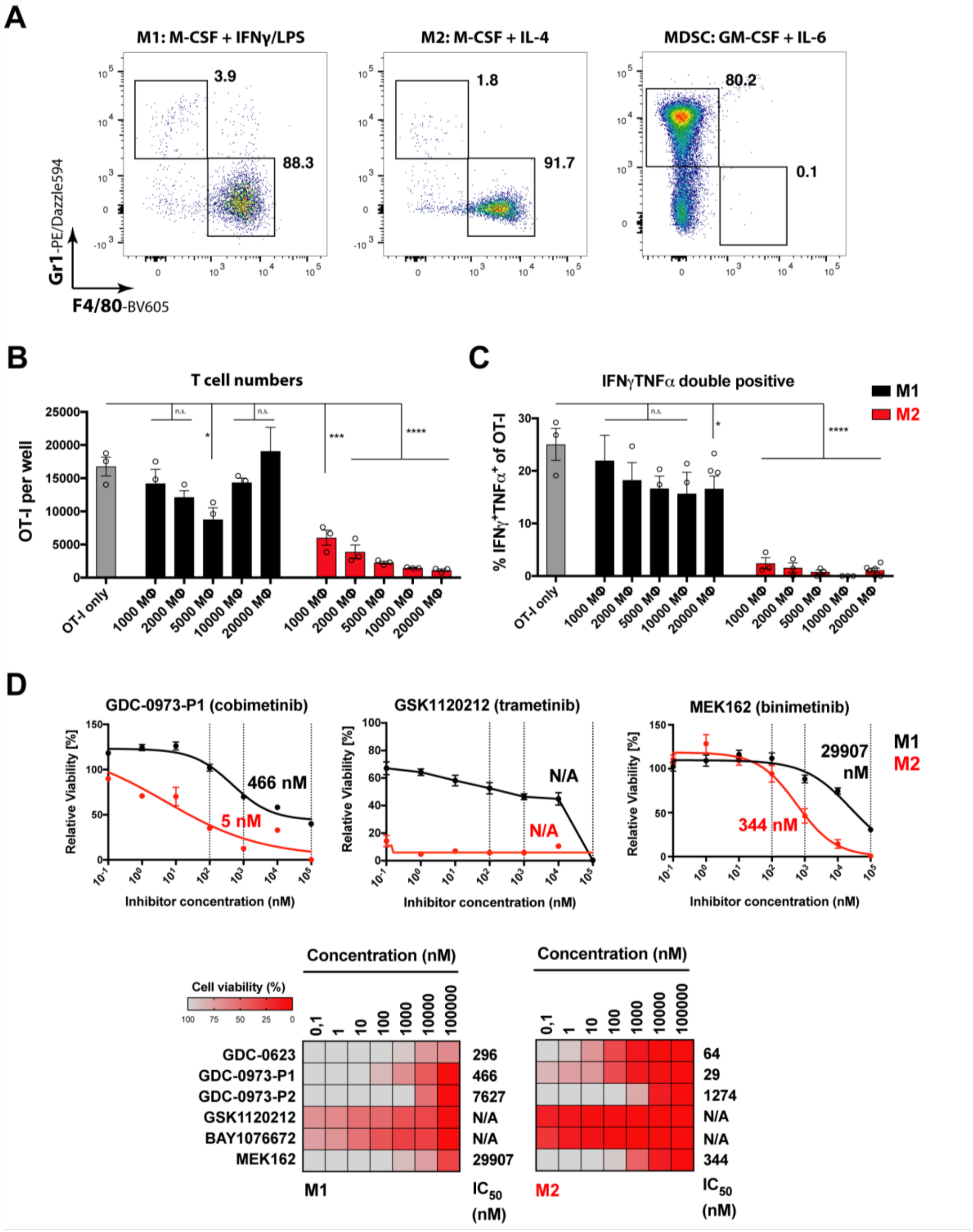

Please see next page for figure legend.

Supplementary Figure 9. M2 type macrophages are sensitive to MEK inhibition. A Flow cytometric analyses of bone marrow-derived monocyte precursors differentiated and polarized into M1- (M-CSF+IFNγ+LPS), M2-like (M-CSF+IL-4) macrophages and MDSCs (GM-CSF+IL-6), respectively. Gated on living, CD11b+ cells and F4/80+ cells for macrophages. **B-C** Macrophage OT-I suppression assay. Monocyte-precursors were isolated from C57BL/6-Ly5.1 mice and cultured in the presence of M-CSF. After seven days of culture, *ex vivo* differentiated macrophages were polarized towards a M1- or M2-like phenotype by addition of IFNγ+LPS or IL-4, respectively. 24 hours after polarization, OT-I T cells (pre-activated for 24 hours in co-cultures with MEC.B7.SigOVA APCs) were added to wells with indicated numbers of M1- or M2-polarized macrophages. On day 4, OT-I numbers (B) were determined by flow cytometry and re-stimulated with 10 µg ml^{-1} SIINFEKL peptide in the presence of GolgiPlug and stained for intracellular IFNγ and TNFα. Mean ± s.e.m, n = 3 (biological replicates from the same experiment), n (20000 macrophages) = 6 (biological replicates from the same experiment). One-way ANOVA with post-hoc Dunnett's test (macrophages vs. OT-I only). Significance levels are indicated by asterisks (* $p \leq 0.05$; ** $p \leq 0.01$; *** $p \leq 0.001$; **** $p \leq 0.0001$). P-values (B): 5000 M1 = 0.0058; 1000 M2: 0.002; 2000-20000 M2: < 0.0001. P-values (C): 20000 M1: 0.0456; 1000-20000 M2 < 0.0001. **D** Representative dose response curves of polarized macrophages treated with indicated MEK inhibitors. Three days after MEK inhibitor addition, cell viability was analyzed by using luminescence cell viability assay kit. Relative cell viability was determined by normalization to the medium control. Mean ± s.e.m, n = 3 (biological replicates from the same experiment).

Supplementary Figure 10

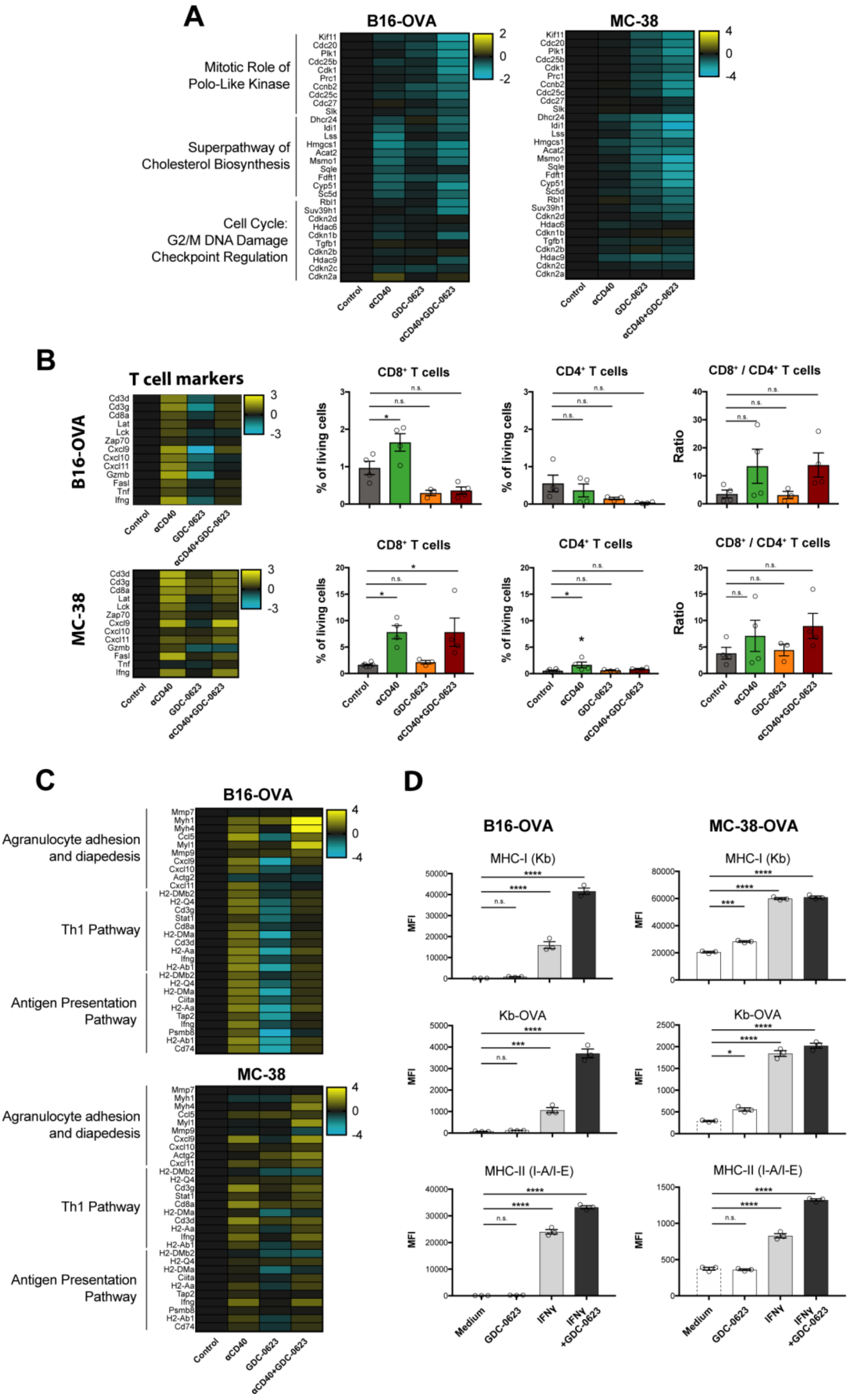

Please see next page for figure legend.

Supplementary Figure 10. Effects of MEKi/CD40 Ab on tumor cell viability and antigen presentation in vivo. A Expression changes of genes, as identified in the PDA30364 model (see Fig. 6), in B16-OVA and MC-38 tumors with focus on downregulated genes; log2 FC. Four mice per treatment group were sacrificed at the end of the MEK inhibitor treatment and subjected to biomarker analyses (B16-OVA: day 18; MC38: day 22 after tumor inoculation). **B** T cell-associated gene expression normalized to control group (log2 FC) and flow cytometric analyses of tumor-infiltrating T cells isolated from B16-OVA and MC-38 tumors. Mean ± s.e.m., n = 4 mice per treatment group. One-way ANOVA with post-hoc Dunnett's test (treatment vs. control). Please note that for the sake of clarity, and to avoid cross-referencing between different figures, we duplicated the presentation of the CD8+ T cell data from Fig. 4C in this figure. P-values CD8, B16-OVA: 0.0331, 0.0533, 0.0590; MC-38: 0.0136, 0.9897, 0.0138; CD4, B16-OVA: 0.7097, 0.2319, 0.0762; MC-38: 0.0214, 0.9923, 0.7054; CD8/CD4, B16-OVA: 0.2419, 0.9999, 0.2134; MC-38: 0.5715, 0.9951, 0.2479. **C** Expression changes of genes identified in the PDA30364 model (see Fig. 6) in B16-OVA and MC-38 tumors with focus on upregulated genes; log2 FC. **D** Flow cytometric analysis of MHC levels on B16-OVA and MC-38-OVA tumor cells treated with 10 ng ml^{-1} IFNγ, 100 nM GDC-0623, combination of IFNγ and GDC-0623 or culture medium. Upper panel MHC-I (H2-Kb), middle panel MHC-I-OVA (H2-Kb-OVA), lower panel MHC-II (I-A/I-E). Mean ± s.e.m, n = 3 (biological replicates from the same experiment). One-way ANOVA with post-hoc Dunnett's test (treatment vs. control). P-values B16-OVA, MHC-I: 0.9399, 0.0001, 0.0001; MHC-I/SIINFEKL: 0.9874, 0.001; 0.0001; MHC-II: 0.9935, 0.0001, 0.0001; MC-38, MHC-I: 0.0001, 0.0001, 0.0001; MHC-I/SIINFEKL: 0.0113, 0.0001, 0.0001; MHC-II: 0.9326, 0.0001, 0.0001. Significance levels are indicated by asterisks (* p ≤ 0.05; ** p ≤ 0.01; *** p ≤ 0.001; **** p ≤ 0.0001).

Supplementary Figure 11

```
#!/bin/sh
# written by: Michael Volkmar
#                  German Cancer Research Center (DKFZ), Dept. D200, Molecular Oncology of
Gastrointestinal Tumors
#            16 February 2018

# get positions in germline exome with sequencing depth of => 30 reads
bedtools2.22.1 genomecov -ibam Germline.bam -g human_genome37.fa -bg > Germline_cov.bg
awk '{ if($4 > 29) { print }}' Germline_cov.bg > Germline_cov30andMore.bg
# Combine covered positions to regions
bedtools2.22.1 merge -i Germline_cov30andMore.bg > Germline_cov30andMore_binned.bg

# get coverage for complete Xenograft exome (mapped to hybrid reference genome)
bedtools2.22.1 genomecov -ibam Xeno.bam -g human_genome37.fa -bga > Xeno_cov.bg

# get overlap of xenograft exome & germline regions with coverage>30
bedtools2.22.1 intersect -a Xeno_cov.bg -b Germline_cov30andMore_binned.bg -wb > \
 Xeno_gl-cov30andMore_overlap.bed

# filter for positions with coverage of 0-1 reads
awk '{ if($4 < 2) { print }}' Xeno_gl-cov30andMore_overlap.bed > Xeno_deleted_pos.bed

# sort BED file and merge deleted positions to regions
bedtools2.22.1 sort -i Xeno_deleted_pos.bed > Xeno_deleted_pos_sorted.bed
bedtools2.22.1 merge -i Xeno_deleted_pos_sorted.bed -c 4 -o mean > Xeno_deleted_regions.bed

# exon-based annotation of deleted regions
bedtools2.22.1 intersect -a Xeno_deleted_regions.bed -b exons-extracted-fromGRCh37.85.bed \
 -wb > Xeno_deleted_regions_annotated.txt
```

Supplementary Figure 11. Custom deletion detection ("deldec") script. This script first extracts all positions from the germline BAM that are sequenced to a depth of ≥30 reads; neighbouring positions are merged to regions. Then, the same procedure is followed for xenograft BAM file without the sequencing depth filtering. This file is subsequently filtered to contain only those regions, which are sequenced to a coverage of ≥30 in the corresponding germline BAM file. From this file, positions are extracted that have a sequencing depth of 0 or 1 reads and can, therefore, be considered somatic deletions. The BED file containing the 'coverage 0-1' positions is sorted and neighbouring positions are merged to regions. To annotate the somatic deletion, the deleted regions BED file is compared to the exon coordinates of GRCh37[3]. Occasionally, instead of somatic deletions, loss-of-heterozygosity (LOH) of deletion SNPs that reside heterozygous in the germline are detected. Also, if sonication regimens during exome library generation and, hence, insert sizes of the germline and xenograft exome libraries are different, regions at the borders of exome capture-targeted regions might be detected as false-positive deletion artefacts.

Supplementary Figure 12

A

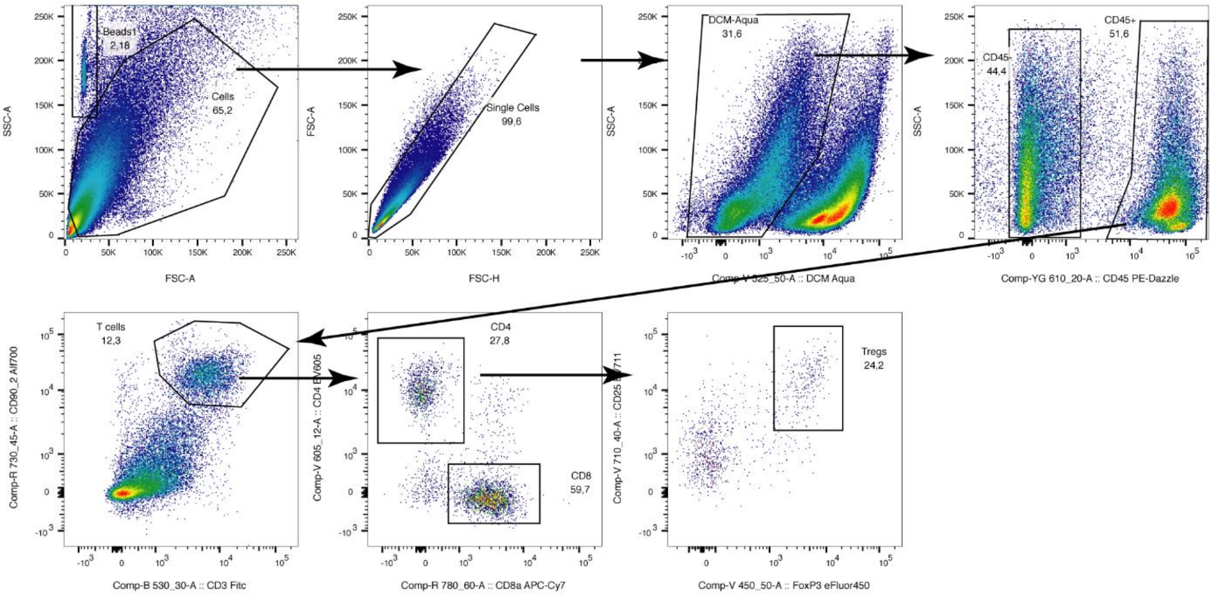

B

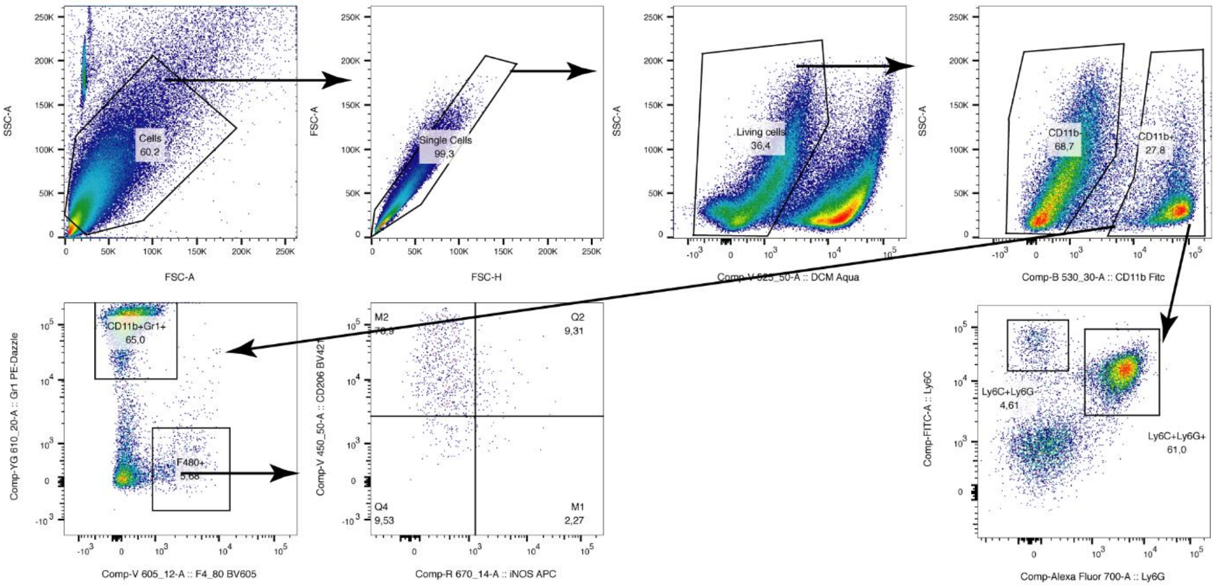

Supplementary Figure 12. Gating strategy for intratumoral T cells (A) and myeloid cells (B).

Supplementary References

1. White AL*, et al.* Interaction with FcgammaRIIB is critical for the agonistic activity of anti-CD40 monoclonal antibody. *J Immunol* **187**, 1754-1763 (2011).

2. White AL*, et al.* Fcgamma receptor dependency of agonistic CD40 antibody in lymphoma therapy can be overcome through antibody multimerization. *J Immunol* **193**, 1828-1835 (2014).

3. Yates A*, et al.* Ensembl 2016. *Nucleic Acids Res* **44**, D710-716 (2016).
