# Supplementary material for: Proimmunogenic impact of MEK inhibition synergizes with agonist anti-CD40 immunostimulatory antibodies in tumor therapy
Source: Nat Commun. 2020 May 1;11:2176. doi: 10.1038/s41467-020-15979-2 (PMC7195409; doi:10.1038/s41467-020-15979-2)
Supplement: Supplementary file 3 — Description of Additional Supplementary Information [file 41467_2020_15979_MOESM3_ESM.pdf]

Description of Additional Supplementary Files

File Name: Supplementary Data 1
Description: Non-synonymous mutations in human PDA cell lines and mouse tumor models as determined by whole exome sequencing.
