# Supplementary material for: Proimmunogenic impact of MEK inhibition synergizes with agonist anti-CD40 immunostimulatory antibodies in tumor therapy
Source: Nat Commun. 2020 May 1;11:2176. doi: 10.1038/s41467-020-15979-2 (PMC7195409; doi:10.1038/s41467-020-15979-2)
Supplement: Supplementary file 5 — Reporting summary [file 41467_2020_15979_MOESM5_ESM.pdf]

nature research

Corresponding author(s): Prof. Dr. Rienk Offringa

Last updated by author(s): Mar 13, 2020

Reporting Summary

Nature Research wishes to improve the reproducibility of the work that we publish. This form provides structure for consistency and transparency in reporting. For further information on Nature Research policies, see Authors & Referees and the Editorial Policy Checklist .

Statistics

For all statistical analyses, confirm that the following items are present in the figure legend, table legend, main text, or Methods section.

n/a	Confirmed	
☐	☒	The exact sample size (*n*) for each experimental group/condition, given as a discrete number and unit of measurement
☐	☒	A statement on whether measurements were taken from distinct samples or whether the same sample was measured repeatedly
☐	☒	The statistical test(s) used AND whether they are one- or two-sided<br>*Only common tests should be described solely by name; describe more complex techniques in the Methods section.*
☒	☐	A description of all covariates tested
☐	☒	A description of any assumptions or corrections, such as tests of normality and adjustment for multiple comparisons
☐	☒	A full description of the statistical parameters including central tendency (e.g. means) or other basic estimates (e.g. regression coefficient) AND variation (e.g. standard deviation) or associated estimates of uncertainty (e.g. confidence intervals)
☐	☒	For null hypothesis testing, the test statistic (e.g. *F*, *t*, *r*) with confidence intervals, effect sizes, degrees of freedom and *P* value noted<br>*Give P values as exact values whenever suitable.*
☒	☐	For Bayesian analysis, information on the choice of priors and Markov chain Monte Carlo settings
☒	☐	For hierarchical and complex designs, identification of the appropriate level for tests and full reporting of outcomes
☒	☐	Estimates of effect sizes (e.g. Cohen's *d*, Pearson's *r*), indicating how they were calculated

Our web collection on statistics for biologists contains articles on many of the points above.

Software and code

Policy information about availability of computer code

Data collection

Microarray analyses with chipster (version 3.11.2):
Dataset name: phenodata.tsv
Created with Chipster 3.11.2
Created with operation: Normalisation / Affymetrix
Parameter Normalization method: RMA
Parameter Stabilize variance: no
Parameter Custom CDF annotation to be used:: Use original Affymetrix annotations
Operation source code:

```
setwd("/opt/chipster/comp/jobs-data/9de20e3d-1748-4545-80e6-98dca8b5ef65/b196e23b-313e-4fa3-919b-45baea4f7701/data")
chipster.tools.path = "/opt/chipster/tools"
chipster.common.path = "../toolbox/tools/common/R"
chipster.module.path = "../toolbox/tools/microarray"
chipster.java.libs.path = "/opt/chipster/shared/lib"
chipster.threads.max = "2"
chipster.memory.max = "8192"
normalization.method <- "rma"
stabilize.variance <- "no"
custom.chiptype <- "empty"
# TOOL norm-affy.R: Affymetrix (Affymetrix preprocessing using CEL-files. Probe sets are automatically flagged using P A M flags where possible. Variance stabilization can be applied only with MAS5 or Plier preprocessing methods. Custom chiptype can't be used with Plier preprocessing.)
# INPUT microarray{...}.cel: microarray{...}.cel TYPE AFFY
# OUTPUT normalized.tsv: normalized.tsv
```

```
# OUTPUT META phenodata.tsv: phenodata.tsv
# PARAMETER normalization.method: "Normalization method" TYPE [mas5: MAS5, plier: Plier, rma: RMA, gcrma: GCRMA, li-wong: Li-Wong] DEFAULT rma (Preprocessing method)
# PARAMETER stabilize.variance: "Stabilize variance" TYPE [yes: yes, no: no] DEFAULT no (Variance stabilazing normalization)
# PARAMETER custom.chiptype: "Custom CDF annotation to be used:" TYPE [empty: "Use original Affymetrix annotations", hgu133ahsentrezg(hgu133a): "hgu133a", hgu133a2hsentrezg(hgu133av2): "hgu133av2", hgu133phsentrezg(hgu133plus): "hgu133plus", hgu133plus2hsentrezg(hgu133plus2): "hgu133plus2", hgu133bhsentrezg(hgu133b): "hgu133b", hthgu133pluspmhsentrezg (hgu133pluspm): "hgu133pluspm", hgfocushsentrezg(hgfocus): "hgfocus", hgu95av2hsentrezg(hgu95av2): "hgu95av2", hgu219hsentrezg (hgu219): "hgu219", moe430ammentrezg(moe430a): "moe430a", moe430bmmentrezg(moe430b): "moe430b", mouse430a2mmentrezg (mouse430a2): "mouse430a2", mouse4302mmentrezg(mouse4302): "mouse4302", mm74av1mmentrezg(mgu74a): "mgu74a", mgu74av2mmentrezg(mgu74av2): "mgu74av2", mgu74bv2mmentrezg(mgu74bv2): "mgu74bv2", mgu74cv2mmentrezg(mgu74cv2): "mgu74cv2", rae230arnentrezg(rae230a): "rae230a", rae230brnentrezg(rae230b): "rae230b", rat2302rnentrezg(rat2302): "rat2302", rgu34arnentrezg(rgu34a): "rgu34a", rgu34brnentrezg(rgu34b): "rgu34b", rgu34crnentrezg(rgu34c): "rgu34c"] DEFAULT empty (The custom CDF annotation to be used. If you choose to use the original Affymetrix annotations, the tool will use the information in the .CEL-files to determine the chiptype. NOTE: If your files are from Gene or Exon array, please use the corresponding normalization tools!)
# JTT 08.06.2006, Created
# JTT 29.06.2006, Changes to column naming on
# JTT 29.01.2007, Changes to phenodata table writing on
# JTT 12.05.2009, Modified to work with R 2.9.0
# MG 23.09.2009, Changes to custom.chiptype PARAMETER to account for changes in naming of custom CDF packages (version 12)
# MG 12.11.2009, Changes to cope with dropped custom package support for certain array types
# MK 25.10.2014, PMA calls created with try-catch. Script polished up.
# EK 12.09.2014, Added HGFocus to altcdfs
# Renaming variables
norm<-normalization.method
stabvar<-stabilize.variance
# Initializes analyses
library(affy)
library(gcrma)
# Reads in data
dat<-ReadAffy()
# Modifies the data objects to take custom chiptype into account
if(custom.chiptype!="empty") {
  chiptype<-custom.chiptype
  library(Biostrings)
  #chiptype<-substr(x=chiptype, start=1, stop=(matchPattern("(", chiptype)@start-1))
  chiptype <- gsub("\\(.*\\)", "", chiptype, perl=T)
  dat@annotation<-chiptype
  dat@cdfName<-chiptype
} else {
  chiptype<-dat@annotation
}
# Since custom annotation packages are no longer supported for hgu133plu
# and for mm74av1 the packages for hgu133plu2 and for mm74av2 are going to
# be used instead
if (chiptype=="hgu133phsentrezg") {
 chiptype <- "hgu133plus2hsentrezg("
}
if (chiptype=="mm74av1mmentrezg") {
 chiptype <- "mm74av2mmentrezg"
}
# Check how many probesets lack mm data. These can be counted by checking rownames
if(norm=="mas5" || norm=="gcrma") {
  if(length(which(is.na(rownames(mm(dat))))) / length(rownames(mm(dat))) > 0) {
    stop("CHIPSTER-NOTE: MAS5 and gcrma methods has not been designed for PM-only arrays. Please use another normalisation method.")
  }
}
# MAS5 normalization
if(norm=="mas5") {
  dat2<-exprs(mas5(dat))
}
# PLIER normalization
if(norm=="plier") {
  library(plier)
  if(length(which(is.na(rownames(mm(dat))))) / length(rownames(mm(dat))) == 0) {
    dat2<-exprs(justPlier(eset=dat,replicate=1:length(dat),get.affinities=FALSE,normalize=FALSE,norm.type=c("together"),augmentation=0.1,defaultaffinity=1.0,defaultconcentration=1.0,attenuation=0.005,seaconvergence=0.000001,seaiteration=3000,gmcutoff=0.15,probepenalty=0.001,concpenalty=0.000001,usemm=TRUE,usemodel=FALSE,fitaffinity=T,plierconvergence=0.00000
```

```
1,plieriteration=3000,dropmax=3.0,lambdalimit=0.01,optimization=0))
    } else {
      dat2<-exprs(justPlier(eset=dat,replicate=1:length(dat),get.affinities=FALSE,normalize=FALSE,norm.type=c
("together"),augmentation=0.1,defaultaffinity=1.0,defaultconcentration=1.0,attenuation=0.005,seaconvergence=0.000001,seaiteration=
3000,gmcutoff=0.15,probepenalty=0.001,concpenalty=0.000001,usemm=FALSE,usemodel=FALSE,fitaffinity=T,plierconvergence=0.00000
1,plieriteration=3000,dropmax=3.0,lambdalimit=0.01,optimization=0))
    }
  }
  # RMA normalization
  if(norm=="rma" & custom.chiptype=="empty") {
    dat2<-exprs(justRMA())
  }
  if(norm=="rma" & custom.chiptype!="empty") {
    dat2<-exprs(rma(dat))
  }
  # GCRMA normalization
  if(norm=="gcrma" & custom.chiptype=="empty") {
    dat2<-exprs(justGCRMA(type=c("fullmodel"), fast=T, optimize.by=c("speed")))
  }
  if(norm=="gcrma" & custom.chiptype!="empty") {
    dat2<-exprs(gcrma(dat))
  }
  # Li-Wong (dChip) normalization
  if(norm=="li-wong") {
    dat2<-exprs(expresso(dat, normalize.method="invariantset", bg.correct=FALSE, pmcorrect.method="pmonly",
summary.method="liwong"))
  }
  # Format data
  dat2<-as.data.frame(round(dat2, digits=2))
  names(dat2)<-paste("chip.", names(dat2), sep="")
  # Apply vsn if normalisation was done using MAS5 or Plier
  if(norm=="plier" || norm=="mas5") {
    if(stabvar=="yes") {
      if(ncol(dat2)<2) {
        stop("CHIPSTER-NOTE: You need to have at least two chip to be able to use VSN!")
      }
      library(vsn)
      dat2<-exprs(vsn(dat2))
    } else {
      dat2<-log2(dat2)
    }
  }
  # Try to create PMA calls
  calls<-try(mas5calls(dat), silent=T)
  if(class(calls) != "try-error") {
    calls<-as.data.frame(exprs(calls))
    names(calls)<-paste("flag.", names(calls), sep="")
    dat2<-data.frame(dat2, calls)
  }
  # Writes out a phenodata table
  sample<-rownames(pData(dat))
  group<-c(rep("", nrow(pData(dat))))
  training<-c(rep("", nrow(pData(dat))))
  time<-c(rep("", nrow(pData(dat))))
  random<-c(rep("", nrow(pData(dat))))
  chiptype<-paste(chiptype, ".db", sep="")
  write.table(data.frame(sample=sample, chiptype=chiptype, group=group), file="phenodata.tsv", sep="\t", row.names=F, col.names=T,
quote=F)
  # Writing out data
  a<-try(library(chiptype, character.only=T))
  if(chiptype!="empty" & class(a)!="try-error") {
    # Including gene names to data
    lib2<-sub('.db','',chiptype)
    symbol<-gsub("\'", "", data.frame(unlist(as.list(get(paste(lib2, "SYMBOL", sep=""))))[rownames(dat2),])
    genename<-gsub("\'", "", data.frame(unlist(as.list(get(paste(lib2, "GENENAME", sep=""))))[rownames(dat2),])
   # Fxes an issue introduced in BioC2.4 where the "#" character is introduced in some gene names
   genename <- gsub("#", "", genename)
   symbol <- gsub("'", "", symbol)
```

```
genename <- gsub("'", "", genename)
# Writes the results into a file
  write.table(data.frame(symbol, description=genename, dat2), file="normalized.tsv", col.names=T, quote=F, sep="\t", row.names=T)
}
if(chiptype=="empty" | class(a)=="try-error") {
  write.table(data.frame(dat2), file="normalized.tsv", col.names=T, quote=F, sep="\t", row.names=T)
}
print("script-finished-succesfully")
```

Custom deletion detection ("deldec") script for exome analysis:

```
#!/bin/sh
# written by: Michael Volkmar
#          German Cancer Research Center (DKFZ), Dept. D200, Molecular Oncology of Gastrointestinal Tumors
#          16 February 2018

# get positions in germline exome with sequencing depth of => 30 reads
bedtools2.22.1 genomecov -ibam Germline.bam -g human_genome37.fa -bg > Germline_cov.bg
awk '{ if($4 > 29) { print }}' Germline_cov.bg > Germline_cov30andMore.bg
# Combine covered positions to regions
bedtools2.22.1 merge -i Germline_cov30andMore.bg > Germline_cov30andMore_binned.bg

# get coverage for complete Xenograft exome (mapped to hybrid reference genome)
bedtools2.22.1 genomecov -ibam Xeno.bam -g human_genome37.fa -bga > Xeno_cov.bg

# get overlap of xenograft exome & germline regions with coverage>30
bedtools2.22.1 intersect -a Xeno_cov.bg -b Germline_cov30andMore_binned.bg -wb > \
 Xeno_gl-cov30andMore_overlap.bed

# filter for positions with coverage of 0-1 reads
awk '{ if($4 < 2) { print }}' Xeno_gl-cov30andMore_overlap.bed > Xeno_deleted_pos.bed

# sort BED file and merge deleted positions to regions
bedtools2.22.1 sort -i Xeno_deleted_pos.bed > Xeno_deleted_pos_sorted.bed
bedtools2.22.1 merge -i Xeno_deleted_pos_sorted.bed -c 4 -o mean > Xeno_deleted_regions.bed

# exon-based annotation of deleted regions
bedtools2.22.1 intersect -a Xeno_deleted_regions.bed -b exons-extracted-fromGRCh37.85.bed \
 -wb > Xeno_deleted_regions_annotated.txt
```

Data analysis

BD FACSDiva Software Version 6.2 (Becton, Dickinson and Company)
FlowJo 10.4.0 (FlowJo LLC)
GraphPad Prism 7.0d (GraphPad Software Inc.)
Ingenuity Pathway Analysis Version 01-14 (Qiagen)
BioAnalyzer 2100 Expert (Agilent Technologies Inc.)
Chipster 3.11.2 (CSC)
Light Cycler480 Software 5.1 (Roche Diagnostics GmbH)
Microsoft Office 2010 & 2016 (Microsoft)
Nanodrop-8000, Version 2.2.1 (Thermo Fisher Scientific)
Unicorn 6.4.1 SP2 (GE Healthcare)
ChemiDoc Touch Imaging System (BioRad)
Vi-CELL XR Cell Viability Analyzer 2.03 (Beckman Coulter GmbH)
Wallac EnVision Manager, Version 1.12 (Perkin Elmer)
ImageLab 6.0.1 (Bio-Rad)

For manuscripts utilizing custom algorithms or software that are central to the research but not yet described in published literature, software must be made available to editors/reviewers. We strongly encourage code deposition in a community repository (e.g. GitHub). See the Nature Research guidelines for submitting code & software for further information.

Data

Policy information about availability of data

All manuscripts must include a data availability statement. This statement should provide the following information, where applicable:

- Accession codes, unique identifiers, or web links for publicly available datasets
- A list of figures that have associated raw data
- A description of any restrictions on data availability

The mouse tumor and in vitro trancriptome data have been deposited in the GEO database under the accession codes GSE144128 (PDA30364: GEM/CD40 tumor), GSE144139 (MC-38: MEKi/CD40 tumor), GSE144145 (PDA30364: MEKi/CD40 tumor), GSE144146 (B16-OVA: MEKi in vitro), GSE144161 (MC-38: MEKi in vitro), GSE144166 (PDA30364: MEKi in vitro), GSE144570 (B16-OVA: MEKi/CD40 tumor) [https://www.ncbi.nlm.nih.gov/gds]. The mouse exome data have been deposited in the ENA database under the accession number ERP119708 [https://www.ebi.ac.uk/ena]. The human exome data have been deposited in the EGA database under the accession code EGAS00001004196 [https://www.ebi.ac.uk/ega/home]. All the other data supporting the findings of this study are available within the article and its supplementary information files and from the corresponding author upon reasonable request. A reporting summary for this article is available as a Supplementary Information file.

Field-specific reporting

Please select the one below that is the best fit for your research. If you are not sure, read the appropriate sections before making your selection.

[x] Life sciences   [ ] Behavioural & social sciences   [ ] Ecological, evolutionary & environmental sciences

For a reference copy of the document with all sections, see nature.com/documents/nr-reporting-summary-flat.pdf

Life sciences study design

All studies must disclose on these points even when the disclosure is negative.

Sample size

For in vitro experiments sample size was determined based on good-laboratory practice (2-3 biological replicates each consisting of 3 technical replicates).
In vivo group sizes were determined on basis of power analyses performed in collaboration with biostatistics department of German Cancer Research Center.

Data exclusions

No data were excluded from the analyses

Replication

In vitro experiments:

- Tumor cytotoxicity assays: At least 2 biological replicates (individual experiments) of each 3 technical replicates were performed. All the replication attemps were succesful. Mean IC50 values are indicated in Fig. 1B and Supplementary Fig. 1C+D.
- Tumor Western Blot analysis: The pERK/ERK/Gapdh WB for the three murine tumor cell lines were performed twice (2 biological replicates with 2 technical replicates each) in presence/absence of MEK Inhibitor GDC-0623.
- Tumor cell cycle analysis: 2/2 successful (first experiment with 10 µM, second experiment with 1 µM); in addition 1 experiment with different MEK inhibitors (GDC-0623, GDC-0973-P1/P2, GSK1120212) with the cell lines PDA30364, MC38, B16F10 (parental). All successful.
- Myeloid cell cytotoxicity assays with GDC-0623 7/7 biological replicates (each 3 technical replicates) successful; with other MEKi 2/2 biological replicares (technical replicates) successful
- Myeloid cell cycle analysis: 3/3 biological replicates (each 3 technical replicates) successful
- Macrophage/OT-I suppression assay: 4/4 biological replicates (each 3 technical replicates) successful
- IFN/MEKi stimulation of tumor cells: 30364-OVA 4/4 biological replicates (each 3 technical replicates) successful; MC38-OVA: 2/2 biological replicates (each 3 technical replicates) successful; B16-OVA 1/1 biological replicate (3 technical replicates) successful
- OT-I proliferation/activation: 2/2 biological replicates (each 3 technical replicates) successful

In vivo experiments:

- OT-I proliferation:
GDC-0623: 6 experiments performed (6/6 successful with each 3-4 mice per treatment group; cumulative data shown in Fig. 2; 3/6 experiment with ex vivo T cell restimulation for IFNg/TNFa analyses).
GDC-0973-P1: 6 experiments performed (6/6 successful with each 3-4 mice per treatment group; cumulative data shown in Fig. 2; 1/6 experiment with ex vivo T cell restimulation for IFNg/TNFa analyses).
GDC-0973-P2: 2 experiments performed (2/2 successful with each 3-4 mice per treatment group; cumulative data shown in Fig. 2; 0/2 experiment with ex vivo T cell restimulation for IFNg/TNFa analyses).
GDC-0941: 4 experiments performed (4/4 successful with each 3-4 mice per treatment group; cumulative data shown in Fig. 2; 1/4 experiment with ex vivo T cell restimulation for IFNg/TNFa analyses).
TEM/GEM: 1 experiment performed with 3 mice per treatment group. No ex vivo restimulation performed.

- OT-I killing assay:
GDC-0623: 3 experiments performed (2/3 successful with 3-4 mice per treatment group; cumulative data shown in Fig. 2). In 1/3 experiment no killing

	- Tumor experiments:<br>For experiments aimed at assessing efficacy of tumor treatment in conjuction with biomarker analyses, groups of at least 6 mice were used. Details on sample size and number of replicates experiments can be found in the individual figure legends.
Randomization	Tumor treatment experiments:<br>Mice were allocated to individual treatment grouped in a semi-randomized fashion by grouping according to initial tumor size so that all treatment groups had a comparable baseline mean tumor volume at treatment start. For all other experiments, e.g. in vivo T cell assays, animals were randomly assigned to different groups.<br><br>Control of other co-variates:<br>Sex: In all experiments more Ly5.1 mice were used.<br>Experimenter: In most experiments, the same experimenter performed the experiment of the same type.<br>Age: All mice had an age of 8-12 weeks<br>Facility: C57BL/6-Ly5.1 (CD45.1+, Ptprca) and NSG (NOD-Prkdcscid) mice were bred in animal facilities of the German Cancer Research Center. OT-I mice (C57BL/6-Ly5.2/CD45.2+; Tg(TcraTcrb)1100Mjb) were purchased from Charles River.<br>Food & Environment: Food and water were provided ad libitum. Mice were maintained on a 12 hour light/dark cycle and environmental enrichment was provided; temperature was main- tained between 20-24°C.<br>Drug cross-contamination: In order to avoid cross-contamination of animals with different small molecule inhibitors and chemotherapy, drugs were allocated to seperate cages.
Blinding	In vitro:<br>Blinding of in vitro experiments was not performed, also because drug solutions were colored differently and not necessary as the final data acquisition was performed by a machine automatically. Where suitable, automatic data analyses was performed, e.g. gating strategy for flow cytometry data and analysis templates for luciferase signal for cytotoxicity experiments. In addition, whenever possible, objective values were used, e.g. mean fluorescence intensity instead of frequency of gated populations.<br><br>In vivo:<br>Blinding of animals during tumor treatment experiments was not performed, because the experiments involved repeated drug dosing and measurement of tumor size performed by the same experimenter. In addition, the drug solution have a different coloring so that blinding was not possible.

Reporting for specific materials, systems and methods

We require information from authors about some types of materials, experimental systems and methods used in many studies. Here, indicate whether each material, system or method listed is relevant to your study. If you are not sure if a list item applies to your research, read the appropriate section before selecting a response.

Materials & experimental systems

n/a	Involved in the study
☐	☒ Antibodies
☐	☒ Eukaryotic cell lines
☒	☐ Palaeontology
☐	☒ Animals and other organisms
☒	☐ Human research participants
☒	☐ Clinical data

Methods

n/a	Involved in the study
☒	☐ ChIP-seq
☐	☒ Flow cytometry
☒	☐ MRI-based neuroimaging

Antibodies

Antibodies used	Fc receptor (FcR) triple block:<br>Consisting of α-CD16/32 clone 2.4G2 (BD Biosciences, cat. #553141), clone 93 (Biolegend, cat. #101302) and α-CD16.2 clone 9E9 (Biolegend, cat. #149502) diluted in FACS buffer (PBS, 200 mM EDTA, 0.5% BSA).<br><br>Cell surface proteins:<br>CD45-PE/Dazzle594 (Biolegend, 1:1000, clone 30-F11, cat. #103145), CD3-FITC (Biolegend, 1:200, clone 17A2, cat. #100204), CD90.2-AF700 (Biolegend, 1:200, clone 20-H12, cat. #105320), CD8a-APC/Cy7 (Biolegend, 1:200, clone 53-6.7, cat. #100714), CD4-BV605 (Biolegend, 1:200, clone RM4-5, cat. #100548), CD25-BV711 (Biolegend, 1:200, clone PC61, cat. #102049), CD279 (Biolegend, 1:200, clone 29F.1A12, cat. #135216), LAG3 (Thermo Fisher, 1:200, clone C9B7W, cat. #17-2231-82), TIM3 (Thermo Fisher, 1:200, clone RMT3-23, cat. #12-5870-82), CD11b-FITC (Biolegend, 1:1000, clone M1/70, cat. #101206), F4/80-BV605 (Biolegend, 1:200, clone BM8, cat.#123133), Gr1-PE/Dazzle594 (Biolegend, 1:1000, clone RB6-8C5, cat. #108452), Ly6G-AF700 (Biolegend, 1:1000, clone 1A8, cat. #127622), Ly6C-FITC (Biolegend, 1:1000, clone HK1.4, cat. #128005), CD40-PE (Biolegend, 1:200, clone 3/23, cat. #124610), I-A/I-E-APC/Cy7 (Biolegend, 1:1000, clone M5/114.15.2, cat. #107627), CD86-PE/Cy7 (Biolegend, 1:1000, clone GL-1, cat. #105014), CD80-BV605 (Biolegend, 1:1000, clone 16-10A1, cat. #104729), H-2Kb-APC (Biolegend, 1:1000, clone AF6-88.5, cat. #116518), H2-Kb/SIINFEKL-PE (Biolegend, 1:1000, clone 25-D1.16, cat. #141603)

Intracellular proteins:
Foxp3-eFl450 (Thermo Fisher, 1:100, clone FJK-16s, cat. #48-5773-82), IFNγ-BV421 (Becton Dickinson, 1:1000, clone XMG1.2, cat. #563376), TNFα-PE (Biolegend, 1:1000, clone MP6-XT22, cat. #506306), CD206-BV421 (Biolegend, 1:200, clone C068C2, cat. #141717), iNOS-APC (Thermo Fisher, 1:200, clone CXNFT, cat. #17-5920-82), Ki67-APC (1:200, clone 16A8, Biolegend, cat. #652406).

Western Blot:
Anti-mouse pERK1/2 (Cell signaling, clone 20G11, cat. #4376, 1:1000), anti-mouse ERK (Cell signaling, clone 137F5, cat. #4695, 1:1000), anti-mouse, Gapdh (GeneTex, cat. #GTX100118, 1:1000), Goat anti-rabbit IgG-HRP (Cell signaling, cat. #7074, 1:3000).

Lots: Different Lots of antibodies were used.

Validation

Triple block: Was performed because myeloid cells in the tumor microenvironment bind antibodies with different Fc receptors that cannot be blocked by one single clone. We used dissociated murine tumor specimens and evaluated single versus triple block for T cell gating. When using triple block, we observed less CD3+/CD90+ dim-med double positive cells. In addition, we used CD11b to gate on lineage negative cells in some experiments.

The following antibodies were validated by FMO controls in tumor tissue, secondary lymphatic organs or using cells from in vitro cultures. In addition, manufacturer's information is provided if existant:
- CD3-FITC (in addition, validated by co-staining of C57BL/6 splenocytes with CD3e performed by the manufacturer (clone 145-2C11, see: https://www.biolegend.com/en-us/search-results/fitc-anti-mouse-cd3-antibody-45)
- LAG3-APC: Validated by manufacturer in CD3/CD28 mouse splenocyte stimulation including isotype control (https://www.thermofisher.com/antibody/product/CD223-LAG-3-Antibody-clone-eBioC9B7W-C9B7W-Monoclonal/17-2231-82)
- F4/80-BV605: FMO staining in tumor and spleen performed. In addition, validated by manufacturer with Thioglycolate-elicited Balb/c mouse peritoneal macrophages stained with F4/80 antibody or isotype control (https://www.biolegend.com/en-us/products/brilliant-violet-605-anti-mouse-f4-80-antibody-8702).
- IFNγ-BV421: FMO staining performed with stimulated OT-I cells in co-culture with MEC.B7.SigOVA-engineered APCs. In addition, validated by manufacturer using PMA/Ionomycin-stimulated mouse splenocytes and isotype control (https://www.bdbiosciences.com/us/applications/research/t-cell-immunology/th-1-cells/intracellular-markers/cytokines-and-chemokines/mouse/bv421-rat-anti-mouse-ifn--xmg12/p/563376).
- TNFα-PE: FMO staining performed with stimulated OT-I cells in co-culture with MEC.B7.SigOVA-engineered APCs. In addition, validated by manufacturer using PMA/Ionomycin-stimulated mouse splenocytes and isotype control (https://www.biolegend.com/en-us/products/pe-anti-mouse-tnf-alpha-antibody-978).
- CD206-BV421: FMO staining of polarized bone marrow derived macrophages in vitro and ex vivo tumor samples. In addition, validated by manufacturer with Thioglycolate-elicited Balb/c mouse peritoneal macrophages stained with CD206 antibody or isotype control (https://www.biolegend.com/en-us/search-results/brilliant-violet-421-anti-mouse-cd206-mmr-antibody-8638).
- iNOS-APC: FMO staining of polarized bone marrow derived macrophages in vitro. In addition, validated by manufacturer with Thioglycolate-elicited mouse peritoneal macrophages stimulated overnight with LPS stained with iNOS antibody or isotype control (https://www.thermofisher.com/antibody/product/iNOS-Antibody-clone-CXNFT-Monoclonal/17-5920-82).
- Ki67-APC: FMO stainings were performed with C57BL/6J splenocytes from tumor-bearing mice and during tumor cell cycle analysis. In addition, validated by manufacturer with Con A+IL-2 stimulated C57BL/6 mouse splenocytes stained with Ki67 antibody and isotype control (https://www.biolegend.com/en-us/products/apc-anti-mouse-ki-67-antibody-8447).
- CD40-PE: FMO stainings were performed with C57BL/6J mouse splenocytes. In addition, mouse BALB/c splenocytes were stained by the manufacturer without information on isotype control (https://www.biolegend.com/en-us/products/pe-anti-mouse-cd40-antibody-4983).
- H-2Kb-APC: FMO stainings were performed with IFNg treated tumor cells. In addition, mouse C57BL/6 splenocytes were stained by the manufacturer without information on isotype control (https://www.biolegend.com/en-us/products/apc-anti-mouse-h-2kb-antibody-6573).
- H2-Kb/SIINFEKL-PE: FMO stainings were performed with IFNg treated tumor cells, including OVA- cells, which served as a negative control. In addition, validated by manufacturer with C57BL/6 mouse splenocytes pulsed with or without SIINFEKL with 25-D1.16 PE or isotype control (https://www.biolegend.com/fr-ch/products/pe-anti-mouse-h-2kb-bound-to-siinfekl-antibody-7247).

Following T cell activation markers were validated using in vitro T cell stimulation (CD3/CD28, PHA and/or PMA/Ionomycin):
- LAG3-APC: Validated by manufacturer in CD3/CD28 mouse splenocyte stimulation including isotype control (https://www.thermofisher.com/antibody/product/CD223-LAG-3-Antibody-clone-eBioC9B7W-C9B7W-Monoclonal/17-2231-82)
- TIM3-PE: We performed mouse splenocytes stimulation with PMA/Ionomycin and PHA to validate the antibody functionality on activated T cells. Here, FMO controls were used. (see also publications with isotype controls: https://www.thermofisher.com/antibody/product/CD366-TIM3-Antibody-clone-RMT3-23-Monoclonal/12-5870-82).

Other following antibodies were not specifically validated as stainings yielded clear and distinct cell populations:
- CD45-PE/Dazzle594: Validated by manufacturer using mouse C57BL/6 splenocytes stained with CD45 antibody without information on isotype control (https://www.biolegend.com/en-us/search-results/pe-dazzle-594-anti-mouse-cd45-antibody-10070).
- CD90.2-AF700: Validated by manufacturer using mouse C57BL/6 thymocytes stained with CD90.2 antibody without information on isotype control (https://www.biolegend.com/de-at/products/alexa-fluor-700-anti-mouse-cd90-2-antibody-3412).
- CD8a-APC/Cy7: Validated by manufacturer using mouse C57BL/6 splenocytes stained with CD8a antibody or isotype control (https://www.biolegend.com/de-at/products/apc-cyanine7-anti-mouse-cd8a-antibody-2269).
- CD4-BV605: Validated by manufacturer using mouse C57BL/6 splenocytes stained with CD4 and CD3 antibody without information on isotype control (https://www.biolegend.com/de-at/products/brilliant-violet-605-anti-mouse-cd4-antibody-7627).

- CD25-BV711: Validated by manufacturer using Con A-stimulated (3 days) C57BL/6 mouse splenocytes stained with CD25. Unstained control was used instead of isotype control (https://www.biolegend.com/de-at/products/brilliant-violet-711-anti-mouse-cd25-antibody-10292).
- CD279: Validated by manufacturer using Con A-stimulated (3 days) C57BL/6 mouse splenocytes stained with CD279 or isotype control (https://www.biolegend.com/en-us/products/pe-cy7-anti-mouse-cd279-pd-1-antibody-7005).
- CD11b-FITC: Validated by manufacturer using using C57BL/6 bone marrow cells stained with CD11b or isotype control antibody (https://www.biolegend.com/en-us/products/fitc-anti-mouse-human-cd11b-antibody-347).
- Gr1-PE/Dazzle594: Validated by manufacturer using C57BL/6 bone marrow cells stained with Gr1 or isotype control antibody (https://www.biolegend.com/en-us/products/pe-dazzle-594-anti-mouse-ly-6g-ly-6c-gr-1-antibody-10319).
- Ly6G-AF700: Validated by manufacturer using C57BL/6 bone marrow cells stained with Ly6G antibody without information on isotype control antibody (https://www.biolegend.com/en-us/products/alexa-fluor-700-anti-mouse-ly-6g-antibody-6754).
-Ly6C-FITC: Validated by manufacturer using C57BL/6 bone marrow cells stained with Ly6C antibody without information on isotype control antibody (https://www.biolegend.com/en-us/search-results/fitc-anti-mouse-ly-6c-antibody-4896).
- I-A/I-E-APC/Cy7: Validated by manufacturer using C57BL/6 mouse splenocytes stained with I-A/I-E and CD11b or isotype control antibody (https://www.biolegend.com/en-us/products/apccyanine7-anti-mouse-i-a-i-e-antibody-5966).
- CD86-PE/Cy7: Validated by manufacturer using mouse C57BL/6 splenocytes stained with CD86 antibody without information on isotype control (https://www.biolegend.com/en-us/products/pe-cy7-anti-mouse-cd86-antibody-3046).
- CD80-BV605: Validated by manufacturer using LPS-stimulated mouse C57BL/6 splenocytes (3 days) stained with CD80 without information on isotype control antibody (https://www.biolegend.com/en-us/products/brilliant-violet-605-anti-mouse-cd80-antibody-7641).
- Foxp3-eFl450: Validated by manufacturer using C57BL/6 mouse splenocytes stained with CD4 and Foxp3 or isotype control antibody (https://www.thermofisher.com/antibody/product/FOXP3-Antibody-clone-FJK-16s-Monoclonal/48-5773-82).

Eukaryotic cell lines

Policy information about cell lines

Cell line source(s)

Human melanoma cell line A375, colon carcinoma cell line Colo205 and B cell leukemia cell line JVM-2 were provided by H. Cerwenka, Immunobiochemistry, UMM Mannheim, Germany. Human mammary carcinoma cell line MDA-MB-231 and hepatocellular carcinoma cell line U-87MG were provided by P. Beckhove, Interventional Immunology, University Regensburg, Germany. Human pancreatic cancer cell line MIA Paca-2 was provided by N. Giese, European Pancreas Center, University Hospital Heidelberg, Germany. Human mammary carcinoma cell line MCF7 were provided by H. Augustin, Vascular Oncology and Metastasis, DKFZ, Heidelberg, Germany. Human mammary carcinoma cell line MDA-MB-468 was purchased from American Type Culture Collection (ATCC). Original source of these cell lines are unknown.

Primary human patient-derived xenograft (PDX) cell lines (TIPC) were generated from human PDA tumor specimen by serial transplantation in NSG mice with subsequent in vitro culturing.

Murine melanoma cell lines B16F10 and B16F10-OVA and murine colon carcinoma cell line MC-38 were provided by Bayer Pharma AG Berlin, Germany. Original source of these cell lines unknown.
The murine pancreatic ductal adenocarcinoma cell line PDA30364 was generated from primary pancreatic tumors of PDA genetically engineered mouse model PDA GEMM Elas-tTA/TetO-Cre Kras+/LSL-G12D Tp53+/LSL-R172H after cessation of doxycycline, which induces DNA recombination, and chronic pancreatitis induced by repetitive injections (three times per week, hourly injection for 6 hours) of 1 µg cholecystokinin analogue cearulein (Sigma). PDA30364-OVA variant was generated via lentiviral transduction of PDA30364 with ecotropic platinum-e retroviral packaging cell line (Cell Biolabs, Inc.) using the pLenti6.3_3xFLAG-Ovalbumin-F2A-EGFP construct (Bayer Pharma AG).

MEC.B7.SigOVA antigen presenting cells were provided by K. Melief, University Medical Centre Leiden, Netherlands (Schoenberger et al., 2003).

Authentication

Human tumor cell lines were authenticated with Multiplex cell line authentication (Multiplexion GmbH, Heidelberg, Germany).
Mouse cell lines were authenticated by whole exome sequencing.

Mycoplasma contamination

All cell lines were tested negative for mycoplasma eith Multiplex Cell Contamination Test (Multiplexion GmbH, Heidelberg, Germany)

Commonly misidentified lines
(See ICLAC register)

Of the cell lines used in this paper, only MCF7 appears on the ICLAC list; our authentication confirmed the correct identity of this cell line.

Animals and other organisms

Policy information about studies involving animals; ARRIVE guidelines recommended for reporting animal research

Laboratory animals	Mice were used in these studies as the least sentient species with an immune system. C57BL/6-Ly5.1 (CD45.1+, Ptprca) and NSG (NOD-Prkdcscid) mice were bred in animal facilities of the German Cancer Research Center. OT-I mice (C57BL/6-Ly5.2/CD45.2+; Tg(TcraTcrb)1100Mjb) were purchased from Charles River. For the experiments male mice were aged between 8-12 weeks. Mice were held in individually ventilated cages in groups of up to five animals. Food and water were provided ad libitum. Mice were maintained on a 12 hour light/dark cycle and environmental enrichment was provided; temperature was main- tained between 20-24°C.
Wild animals	Wild animals are not involved in the study
Field-collected samples	Wild animals are not involved in the study
Ethics oversight	All animal procedures followed the institutional laboratory animal research guidelines and were approved by the governmental authorities (Regional Administrative Authority Karlsruhe, Germany).

Note that full information on the approval of the study protocol must also be provided in the manuscript.

Flow Cytometry

Plots

Confirm that:

- [x] The axis labels state the marker and fluorochrome used (e.g. CD4-FITC).
- [x] The axis scales are clearly visible. Include numbers along axes only for bottom left plot of group (a 'group' is an analysis of identical markers).
- [x] All plots are contour plots with outliers or pseudocolor plots.
- [x] A numerical value for number of cells or percentage (with statistics) is provided.

Methodology

Sample preparation	Tumor tissue (50-200 mg) was digested using a human tumor dissociation kit (Miltenyi) according to manufacturer's instructions in conjunction with the gentleMACS Octo tissue dissociator (Miltenyi) with the program '37C_h_TDK_3'. After enzymatic digestion and homogenization, tumor cell suspensions were poured through a 100 µm pre-coated with 3% BSA/PBS. Spleens were isolated and mashed through a 100 µm cell strainer. Isolated splenocytes were resuspended in ACK lysis buffer (Lonza) in order to lyse red blood cells. Live-dead discrimination was performed with Zombie Aqua dead cell marker (Thermor Fisher). After an incubation period of 10 min at 4 °C, cells were washed twice in FACS buffer and resuspended 1:100 Fc receptor (FcR) triple block, consisting of α-CD16/32 clone 2.4G2 and clone 93 as well as α-CD16.2 clone 9E9 diluted in FACS buffer (PBS, 200 mM EDTA, 0.5% BSA). After 10 min blocking, extracellular staining was performed. After washing and centrifugation, pelleted cells were resuspended in antibody mixes and incubated at 4 °C for 25 min. In case of staining of intracellular antigens, cells were fixed using the Transcription Factor Buffer set (BD) according to the manufacturer's instruction. Intracellular antibodies were diluted in perm-wash buffer. In order to monitor the effector cytokine production of TILs, single cells suspensions were generated as described above and incubated in T cell medium containing 1:1000 dilution of Golgi-Plug for 5 hours at 37°C supplemented with 100 ng/ml PMA 500 ng/ml Ionomycin. Cells were subsequently stained for T cell markers and intracellular effector cytokines IFNγ and TNFα. For cell cycle analyses, cells were treated with small molecule inhibitors or DMSO and incubated at 37°C, 5% CO2 for 72h, detached by addition 0.25% trypsin-EDTA and resuspended in 200 µl ice cold (pre-chilled at -20°C) 70% ethanol for fixation for 2 hours at -20°C. After fixation, cells were rinsed with FACS buffer and stained with Ki67 antibody for 30 min at RT. After this step, cells were washed with FACS buffer and resuspended in PI staining solution (50 µl PI + 12.5 µl RNAse + 16 µl MgCl2 in PBS). 20 min after incubation at RT samples were recorded with a BD LRS Fortessa flow cytometer.
Instrument	BD LSR Fortessa Cell Analyzer
Software	BD FACSDiva Software
Cell population abundance	Cell population abundance in the different types of experiments was as follows:<br>1. In vitro experiments with OT-I T-cells isolated through enrichment from spleens of OT-I TCR-transgenic mice, followed by coculture with adherent MEC.B7.SigOVA-cells<br>Non-adherent cell population isolated from culture:<br>• CSFE-labeled OT-I T-cells: >95%<br><br>2. In vivo immunization experiments with OT-I T-cell infused mice<br>Whole mouse splenocyte population consisting of:<br>• CD45.1+ recipient splenocytes: >98%<br>• CFSE-labeled, CD45.2+ OT-I T-cells: 0.1-2%, depending on experimental outcome<br><br>3. Whole tumor flow cytometric analysis

Enzymatically dissociated tumor cell isolate
- CD45-negative cell fraction:
  - B16-OVA: 30-99%
  - MC-38: 20-50%
  - PDA30364: 40-80%
- CD45+ cell fraction:
  - B16-OVA: 0.5-20%
  - MC-38: 50-80%
  - PDA30364: 20-60%
- CD3+ T-cell fraction within CD45+ fraction:
  - B16-OVA: 15-60%
  - MC-38: 6-40%
  - PDA30364: 4-40%
- CD11b+ myeloid fraction within living cell fraction:
  - B16-OVA: 2-30%
  - MC-38: 20-50%
  - PDA30364: 10-60%

4. In vitro experiments with M1-type or M2-type macrophages or MDSCs generated from mouse bone marrow precursor cells
- CD11b+ cells: >90%
- F4/80+ cells within CD11b+: 80-90%, depending on experimental outcome
- Gr1+ cells within CD11b+: 80-90%, depending on experimental outcome

Gating strategy

Numbers refer to experiment types as listed above.
1. In vitro experiments with OT-1 T-cells isolated through enrichment from spleens of OT-1 TCR-transgenic mice, followed by coculture with adherent MEC.B7.SigOVA-cells
All cells: SSC-A/FSC-A
Single cells: FSC-A/FSC-H
Living cells: Dead cell marker negative cells
OT-I: CD45.2 positive, CD45.1 negative cells

2. In vivo immunization experiments with OT-1 T-cell infused mice
All cells: SSC-A/FSC-A
Single cells: FSC-A/FSC-H
Living cells: Dead cell marker negative cells
OT-I: CD45.2 positive, CD45.1 negative cells

3. Whole tumor flow cytometric analysis
All cells: SSC-A/FSC-A
Single cells: FSC-A/FSC-H
Living cells: Dead cell marker negative cells
All leukocytes: CD45 positive cells

All T cells: CD11b negative; CD3CD90.2 double positive
CD8 T cells: CD8 positive, CD4 negative cells
CD4 T cells: CD8 negative, CD4 positive cells
Regulatory T cells: CD4 positive, CD25 positive, Foxp3 positive cells
OT-I T cells: CD45.2 positive, CD45.2-negative, CD3 CD90.2 CD8 positive

All myeloid cells: CD11b positive cells
MDSC: CD11b positive, Gr1 positive, F4/80 negative cells
PMN-MDSCs: CD11b positive, F4/80 negative, Ly6G positive, Ly6C negative cells
M-MDSCs: CD11b positive, F4/80 negative, Ly6G negative, Ly6C positive cells
Macrophages: CD11b positive, F4/80 positive, Gr1 negative cells
M2-like macrophages: CD11b positive, F4/80 positive, CD206 positive cells
M1-like macrophages: CD11b positive, F4/80 positive, iNOS positive cells

4. In vitro experiments with M1-type or M2-type macrophages or MDSCs generated from mouse bone marrow precursor cells
All cells: SSC-A/FSC-A
Single cells: FSC-A/FSC-H
Living cells: Dead cell marker negative cells
All myeloid cells: CD11b positive cells
MDSC: CD11b positive, Gr1 positive, F4/80 negative cells
Macrophages: CD11b positive, F4/80 positive, Gr1 negative cells

☒ Tick this box to confirm that a figure exemplifying the gating strategy is provided in the Supplementary Information.
